# Tropical Tree Branch-Leaf Nutrient Scaling Relationships Vary With Sampling Location

**DOI:** 10.3389/fpls.2019.00877

**Published:** 2019-07-05

**Authors:** Demetrius Lira-Martins, Emma Humphreys-Williams, Stanislav Strekopytov, Francoise Yoko Ishida, Carlos Alberto Quesada, Jon Lloyd

**Affiliations:** ^1^Department of Life Sciences, Imperial College London, Ascot, United Kingdom; ^2^Imaging and Analysis Centre, Natural History Museum, London, United Kingdom; ^3^Centre for Tropical, Environmental and Sustainability Sciences, College of Science and Engineering, James Cook University, Cairns, QLD, Australia; ^4^Coordination of Environmental Dynamics, National Institute for Amazonia Research, Manaus, Brazil; ^5^Faculdade de Filosofia, Ciencias e Letras de Ribeirão Preto, Universidade de São Paulo, Ribeirão Preto, Brazil

**Keywords:** traits, wood density, nutrients, potassium, soils, climate, mixed model, ecological fallacy

## Abstract

Bivariate relationships between plant tissue nutrient concentration have largely been studied across broad environmental scales regardless of their covariation with soil and climate. Comparing leaf and branch wood concentrations of C, Ca, K, Mg, N, Na, and P for trees growing in tropical forests in Amazonia and Australia we found that the concentrations of most elements varied with sampling location, but with foliar and branch woody tissues varying from site to site in different ways. Using a Mixed Effect Model (MEM) approach it was further found that relationships between branch and leaf concentrations within individual plots differed in terms of both slope and/or significance to the ordinary least squares (OLS) estimates for most elements. Specifically, using MEM we found that within plots only K and Mg were correlated across organs, but with the K cross-organ intercept estimates varying significantly between sites. MEM analyses further showed that within-plot wood density variations were also negatively related to wood K and Na, suggesting a potentially important role for these cations in water transport and/or storage in woody tissues. The OLS method could not detect significant correlations in any of the above cases. By contrast, although Ca, N, and P leaf and wood tissue concentrations showed similar patterns when individual elements were compared across sites, MEM analyses suggested no consistent association within sites. Thus, for all these three elements, strong within-tree scaling relationships were inferred when data were analyzed across sites using OLS, even though there was no relationship within individual sites. Thus (as for Ca, N, and P) not only can a pooling of data across sites result in trait (co)variations attributable to the environment potentially being incorrectly attributed solely to the species and/or individual (the so-called “ecological fallacy”), but in some cases (as was found here for K and Na) the opposite can also sometimes occur with significant within-site covariations being obscured by large site-site variations. We refer to the latter phenomenon as “environmental obfuscation.”

## Introduction

Most terrestrial plants acquire the bulk of the mineral nutrients required for growth and reproduction from their supporting soil substrate. Hence, plant nutrient concentrations should to at least some extent reflect the soil nutrient status (Vitousek, 1984). To date, most analyses of variations in plant nutrient status have concentrated on differences in foliage concentrations and with the bulk of these analyses focussing on nitrogen and/or phosphorus (Reich and Oleksyn, 2004; Wright et al., 2004; Niklas, 2006; Townsend et al., 2007; Ordoñez et al., 2009; Hidaka and Kitayama, 2011), and with only some studies also including cations such as calcium, magnesium, and potassium (Fyllas et al., 2009; Lloyd et al., 2010; Asner and Martin, 2016).

Despite the common use of leaves as indicators of plant nutrient status and ecosystem processes, wood also plays a crucial role in individual plant and ecosystem nutrient balance. At the individual level, woody tissues can serve as a storage for nutrient re-translocated from leaf resorption (Weatherall et al., 2006), also providing nutrients for cambial activity (Fromm, 2010). Living cells within the woody stem such as parenchyma and/or septate fibers presumably also require essential elements for their day-to-day function as is the case for other living plant tissues. There is also some evidence that certain cations, such as potassium, may play a specific and important role in facilitating the refilling of cavitated xylem vessels in the xylem (Trifilò et al., 2014).

Although for most nutrients observed concentrations within woody tissues are typically found at less than one-tenth those of the foliage (Proctor, 1989; Heineman et al., 2016), for a mature forest the woody biomass can be as much as 50 times greater than that of the foliage (Vitousek and Sanford, 1986; Lloyd and Farquhar, 1996). Thus, at the ecosystem level, woody tissues may account for a considerably greater proportion of the total plant nutrients than do the leaves, and with above-ground nutrient stocks sometimes even exceeding the total amount available in the soil (Johnson et al., 2001). Indeed, it has been estimated that in some forests, 90% of the total Ca, K, P, and N ecosystem pool may be tied up in aboveground woody stocks, with this proportion being lower if deeper soil layers are considered (Bond, 2010).

From what limited data is available, variations in woody tissue nutrient concentrations are broadly reflective of differences in soil nutrient status (Schreeg et al., 2014; Heineman et al., 2016) and simple site-independent scaling relations of nutrient concentrations across branch wood and foliar organs have been proposed (Yang et al., 2014; Heineman et al., 2016; Yan et al., 2016). Yet, it is also clear for leaves at least, that soils and climate may affect nutrient concentrations in complex ways, and with the nature of any covariances not necessarily the same when it is genotype rather than environment which is the major source of variation (Fyllas et al., 2009; Schrodt et al., 2015; Turnbull et al., 2016). It is thus by no means obvious that when compared across a range of sites of different climatic and/or edaphic characteristics that unambiguous scaling relationships between leaf and stem nutrient concentrations should necessarily hold.

Here, specifically addressing the question as to whether within-community leaf/wood nutrient relationships vary independently of sampling location, we examine the relationships between leaf and branch wood nutrients across eight moist tropical forest plots (six in the Amazon Basin and two in Far North Queensland, Australia) with sites deliberately chosen to reflect a wide range of soil chemistries and climate. We use a multi-level modeling (MLM) framework to account the possibility that variations in sampling location may influence trait inter-relationships in a different way than when different trees growing at the same location are compared. As well as comparing relationships between concentrations of carbon, calcium, magnesium, potassium, nitrogen, sodium, and phosphorus in wood vs. leaves we also examine the relationship between both leaf and wood nutrient concentrations and stem wood density (ρ), which is considered a key structural trait associated with a range of woody plant life history strategies (Muller-Landau, 2004; Chao et al., 2008; Poorter et al., 2008; Kraft et al., 2010).

**FIGURE 1:**
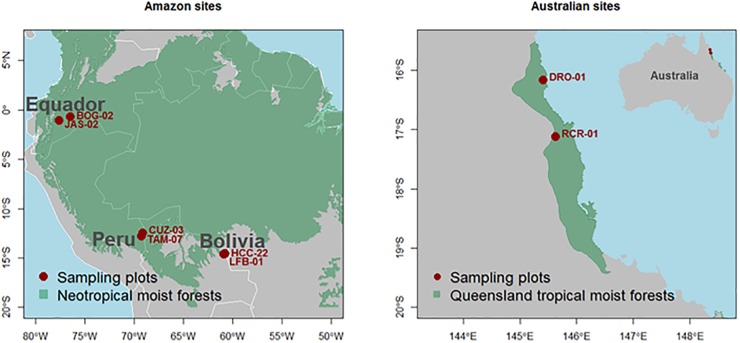
Locations of the eight sample plots.

## Materials and Methods

### Study Sites and Sampling

Of the 82 Amazon Basin forest plots described in Patiño et al. (2009) and Fyllas et al. (2009), two plots of contrasting soil chemical properties but similar climatic conditions were selected in each of Bolivia, Ecuador, and Peru. For the plot selection, we aimed to maximize soil nutrient status variability and control for soil physical properties, hence all plots represented non-flooded forests. We controlled for climatic variation within country by selecting plots with similar annual precipitation, but with our experimental design also incorporating significant precipitation variation between countries. For all the Amazon forest plots, leaf nutrient, and wood density data were already available from Patiño et al. (2009) and Fyllas et al. (2009), but with new data collected from two tropical forest plots located in Far North Queensland, Australia also collected as part of the study (Figure 1). For each plot pairing, mean annual precipitation (*P*_A_) were broadly similar, ranging from 1.45 m a^−1^ in Bolivia to 3.20 m a^−1^ in Australia. Throughout the text we use the terms “low” and “high” to describe the contrasting soil nutrient status within each country as evaluated by the total sum of exchangeable bases as measured to 0.3 m depth (Table 1).

**Table 1:** Mean annual temperature (*T*_A_), mean annual precipitation (*P*_A_), altitude, and location for each plot where leaf and wood were sampled.

Country	Plot	Soil cation status	*T*_A_ (°C)	*P*_A_ (m)	Altitude (m)	Longitude	Latitude
Australia	RCR-01	Low	20.9	1.96	710	145.63	−17.12
	DRO-01	High	24.2	3.20	90	145.41	−16.17
Bolivia	LFB-01	Low	24.1	1.45	299	−60.83	−14.58
	HCC-22	High	21.5	1.51	637	−60.73	−14.53
Ecuador	JAS-02	Low	23.9	3.71	431	−77.62	−1.07
	BOG-02	High	24.9	3.17	261	−76.47	−0.70
Peru	TAM-07	Low	25.4	2.46	218	−69.26	−12.83
	CUZ-03	High	25.3	2.08	203	−68.96	−12.50

#### Amazon Basin

Descriptions of leaf sampling methods have already been given in Fyllas et al. (2009) and with wood sampling methods described in Patiño et al. (2009) and are thus repeated only briefly here. Leaf and wood samples were taken from upper branches of approximately 20 trees with diameter at breast height (*D*) greater than 10 cm in each plot (Supplementary Table S1). For each collected branch, 10–20 fully developed and healthy leaves were randomly chosen for the quantification of leaf elemental concentrations (Fyllas et al., 2009). From the same branches, wood samples of 5 to 10 cm long and 1 to 2 cm diameter had been trimmed for wood density (ρ) measurements (Patiño et al., 2009). With bark and phloem removed from the wood samples, the fresh volume of each wood sample was then calculated as the length of the sample divided by the average diameter of two perpendicular measurements at each end of the sample. Many studies have used similar sized material for an evaluation of the relationship between ρ and leaf traits of tropical trees (Santiago et al., 2004; Meinzer et al., 2008) as well as xylem resistance to cavitation and mechanical strength (Hacke et al., 2001; Jacobsen et al., 2007a,b; Pratt et al., 2007).

All wood density samples were dried at 90°C until constant mass (three to 4 days) and with ρ calculated as the dry mass (g) divided by the fresh volume (cm^3^). Using the dried samples still available from the Patiño et al. (2009) study, laboratory analyses for wood nutrient contents were undertaken as described below.

#### Australia

Leaf and branch samples were collected during June/July 2015. One site (RCR-01) was in Atherton Tablelands next to the Robson Creek 25 ha permanent plot (Bradford et al., 2014) with the second site (DRO-01) located in Daintree National Park World Heritage Area (Laidlaw et al., 2007). For the RCR-01 site, sampling around the 25-ha permanent plot was undertaken using a cherry picker to access the tree canopy. For the DRO-01 site, we used a canopy crane that is available and managed by James Cook University. The methods for sampling the leaf and branch material in the Australian plots were as for the South American plots as described in the Section “Amazon Basin” and with the species sampled listed in Supplementary Table S1.

### Nutrient Determinations

#### Foliar Nutrients

Leaf chemical analysis procedures for the pre-existing data from the Bolivia, Ecuador, and Peru plots have already been described in detail in Lloyd et al. (2010) and are thus only briefly repeated here. For Ca, K, Mg, Na, and P determinations, about 100 mg of leaf material was digested in a microwave (Multiwave, Anton Paar) with the addition of 3 ml of 65% HNO_3_, after which the extract was analyzed by inductively coupled plasma optical emission spectroscopy (ICP-OES) (Optima 3300 DV, Perkin Elmer). For C and N determinations, 15–30 mg of leaf material was analyzed using a Vario EL elemental analyzer (Elementar Analysensysteme). All analyses had been carried out in the Central Analytical and Stable Isotope Facilities at the Max-Planck Institute for Biogeochemistry in Jena, Germany. The analysis of leaf material from Australia follows the methods as described for wood material in the Section “Wood Nutrients” directly below.

#### Wood Nutrients

Branch wood material was dried for 48 h at 60°C. The material was ground in a Planetary Ball Mill (Retsch PM 400) and for Ca, K, Mg, Na, and P determinations, 200 mg of the finely ground material was digested using a microwave system (CEM MARS X) in XP1500 Plus fluoropolymer vessels with 5 ml of concentrated nitric acid and 0.5 ml of hydrogen peroxide (both trace element analysis grade). Certified reference material of Willow wood (WEPAL-IPE-220) was run every three batches. The extracts were then diluted to 50 ml in ultrapure water and then analyzed using ICP-OES (Thermo iCap 6500 Duo). For all elements analyzed the measured values for this reference material were within the uncertainty of the certificate values. Carbon and nitrogen analyses were undertaken by using a CHN elemental analyzer Vario EL. For the latter, 5 mg of wood material was used and each sample had three replicates for each measurement. A range of plant quality control samples was used to check the accuracy of the C and N analysis. The ICP-OES and C and N analyses were undertaken at the Imaging and Analysis Centre at the Natural History Museum in London, United Kingdom with the digestions having been being carried out at Imperial College London – Silwood Park Campus, United Kingdom. Although the extraction and analysis procedures were slightly different for the previously determined Amazon leaf nutrient determinations as opposed to the Australian foliar and Amazon/Australian wood nutrient determinations undertaken specifically as part of this study, these procedural differences should have had minimal effects on the elemental concentrations presented (Wheal et al., 2011).

### Soil and Climate Data

Data on soil physical and chemical properties for the six Amazon plots are as in Quesada et al. (2010) with all analyses having been undertaken at the Max-Planck Institute for Biogeochemistry in Jena, Germany. For Australia, data from DRO-01, obtained using the same methodology comes from the University of Leeds and is as presented in Veenendaal et al. (2015). Briefly, six to eight soil samples were taken from each site and with average values 0–30 cm depth presented here. Soil nitrogen was determined by using an elemental analyzer (Pella, 1990), exchangeable cations using the silver thiourea method (Pleysier and Juo, 1990) and total P by digestion of soil samples by using sulphuric acid and hydrogen peroxide. The particle size distribution was determined by the Boyoucos method (Gee and Bauder, 1986). Soil data from RCR-01 has been provided by the Australian Terrestrial Ecosystem Research Network, with soil N determined by using an elemental analyzer (Leco), exchangeable cations extracted by using a mixture of 0.1 M NH_4_Cl and BaCl_2_, total P determined by soil digestion in H_2_SO_4_ in the presence of a BDH Kjeldahl catalyst tablet (Rayment and Lyons, 2011), and with particle size determined using the “pipette method” (Indorante et al., 1990). Although the procedures used for the RCR-01 soils were slightly different to those employed for the other five sites, in all cases the measurements should be directly comparable, with for example both total P extraction approaches obtaining only “pseudo-totals” and with both cation extraction methods specifically for the variable charge soils of humid tropical regions (Rayment and Lyons, 2011). Likewise, both the Boyoucos and pipette methods for soil particle size determination are well known to give very similar results (Day, 1965).

Climate data for all plots have been obtained from the interpolated WorldClim dataset at 2.5 min spatial resolution (Hijmans et al., 2005). These were total annual precipitation *P_A_* and mean annual temperature *T_A_*.

### Statistical Analyses

#### Effects of Site Cation Status on the Measured Traits

For comparisons of the overall arithmetic mean between high and low soil cation status plots ANOVA models were used. All data were log_10_ transformed, with the location where samples were taken used as a covariate to improve statistical power.

#### Redundancy Analysis

For an evaluation of the relative influences of the measured soil and climate factors as modulators of the observed variation in wood and leaf traits, we employed redundancy analysis (RDA). For the RDA, which is essentially a multiple response linear regression followed by a principal component analyses (PCA) of the table of the fitted values (Rao, 1973), the response variables examined were the wood and leaf elemental concentrations and wood density. For the explanatory variables (the number of which, with eight sites forming part of the study, was necessarily limited to a maximum of seven), we took soil exchangeable Ca, K, Mg, and Na and soil total P as being the most likely representatives of overall soil properties and with total annual precipitation (*P_A_*) and mean annual temperature (*T_A_*) taken as the climate variables. This analysis was undertaken using the vegan package (Oksanen et al., 2019) with results presented as a triplot using Type–II scaling where the eigenvectors are scaled to the square root of their eigenvalue (Legendre and Legendre, 1998).

#### Multilevel Modeling

The structure of the sampling design can be considered to represent a multilevel set-up in which every tree (level-1) is located within a specific plot (level-2). Due to there being only four geographical locations (viz. countries) in the sampling design, we did not include geographic location as a higher level in the analysis. Rather, we tested whether potential derived plot effects and/or average trait values in each plot were correlated with plot-dependent soil and/or climate variables, which is considered to potentially better allow for an informative understanding of the underlying factors affecting trait associations (Fyllas et al., 2009). Further, as species replication both within and across plots was minimal (Supplementary Table S1) we did not attempt to separate the between-tree variation into an intra- vs. inter-specific component. Rather, we simply fitted a mixed effect model (MEM) examining the relationship between the wood and leaf traits within different trees as modulated by the plot location of the form:

(1) $$W_{\mathrm{tp}}=\gamma_{00}+\gamma_{10}L_{\mathrm{tp}}+U_{0p}+R_{\mathrm{tp}},$$

where *W_tp_* represents some wood trait (*W*) sampled from tree *t* located within plot *p*, *L* refers to foliar traits as determined on the same tree; γ_00_ is the overall intercept; γ_10_ is the overall regression coefficient describing the relationship between wood and leaf traits; *U*_0p_ is a random variable which measures the difference between the average value of the studied trait at plot *p* and the average trait in the whole dataset (γ_00_), i.e., the plot effect, thereby controlling for the effect of variations between plots, and with *R*_tp_ representing the residual error. The *R*_tp_ and *U*_0p_ terms are assumed to be drawn from normally distributed populations (log_10_ transformed) and the variance of the first level residuals (*R*_tp_) is assumed to be constant. We also analyzed the relationship of ρ with elemental concentrations in both wood and leaf by modeling ρ as an independent variable (i.e., as *W*_tp_) in equation (1).

All MEM model fits were carried out using the lme4 package (Bates et al., 2015) available within the R (3.4.3) statistical platform (R Core Team, 2018). Associated probability values were extracted using the lmerTest package (Kuznetsova et al., 2017) and to assess the significance of the random intercept term a restricted likelihood ratio test was implemented using the function exactLRT from package RLRsim (Scheipl et al., 2008). This test simulates values from a finite distribution of simultaneous tests thereby providing an exact likelihood ratio test.

Because observed associations might be simply due to average differences between groups (i.e., plots), we also tested for the significance of plot mean differences for each inter-organ trait association. The model for this evaluation was of the form:

(2) $$W_{\text{tp}}=\gamma_{00}+\gamma_{10}L_{\text{tp}}+\gamma_{01}{\bar{L}}_{0p}+U_{0p}+R_{\text{tp}},$$

where γ_01_ refers to the between-plot coefficient which describes how *W* varies with ${\bar{L}}_{0\text{p}}$ (viz. the mean leaf trait value at the plot level as designated by the subscript). Hence, we employed models of between-site association, as well as within site association, including the mean plot values as a predictor. As discussed in Snijders and Bosker (2012), when analyzed in this way, the within-site regression coefficient quantifies the slope between the association of the assessed traits within a given site, whereas the between-site regression coefficient expresses the slope of the association between the mean site values for the two traits.

Equations 2 and 3 are effectively “random intercept models” for which the log-log slope (but not the intercept) of the relationship is considered the same across all plots and with the validity of this assumption checked by the function exactLRT from RLRsim.

We also employed ordinary least squares (OLS) models which have been more widely used to evaluate these bivariate relations and compared the model predictors with the mixed model (MEM) estimates. These OLS models were of the form:

(3) $$W_{\mathrm{tp}}=\beta_{0}+\beta_{1}L_{\mathrm{tp}}+\varepsilon ,$$

where β_0_ represents the overall intercept of the evaluated relation, β_1_ is the coefficient of association between leaf and wood traits across all sites and ε is the residual error.

## Results

### Soil Properties

Soil physical and chemical characteristics of the study sites are detailed in Table 2. Exchangeable calcium, [Ca]_ex_ varied by over two orders of magnitude ranging from 0.20 mmol kg^−1^ (LFB-01 in Bolivia) to 59.8 mmol kg^−1^ (BOG-02 in Ecuador). On the other hand, exchangeable potassium, [K]_ex_, showed a much more constrained variation with the highest concentration of 2.4 mmol kg^−1^ (RCR-01 in Australia) just more than three times higher than the lowest values of 0.7 mmol kg^−1^ found at the Bolivian LFB-01. Exchangeable magnesium, [Mg]_ex_, ranged from 0.60 mmol kg^−1^ (LFB-01 in Bolivia) to 16.7 mmol kg^−1^ (CUZ-03 in Peru). The two Australian plots had the highest soil exchangeable sodium, [Na]_ex_, of 0.50 mmol kg^−1^ for RCR-01 and 0.60 mmol kg^−1^ for DRO-01 with these values being fifty times greater than LFB-01 (0.01 mmol kg^−1^). The variation in C:N ratio was constrained across plots with a twofold increase from the Peruvian CUZ-03 (7.6) to Australian DRO-01 (16.9). Total soil phosphorus [P]_t_ had the highest value of 23.5 mmol kg^−1^ for CUZ-03 in Peru being seven times greater than the lowest 3.3 mmol kg^−1^ found at LFB-01.

Overall our plot cation status categorization (viz. “high” vs. “low”) reflects differences in overall soil nutrient status reasonably well, although with some exceptions of note. For example, nitrogen was higher in the “low” soil cation status plot of Ecuador than for the “high” cation status soil, and exchangeable potassium [K]_ex_ was higher for the “low” versus the “high” cation status plot in Australia. Soil exchangeable sodium [Na]_ex_ was also slightly higher for the “low” vs. “high” cation status plots of Ecuador and Peru.

**Table 2:** Soil classification according to World Reference Bases for soil resources **(Quesada et al., 2011)**, soil textural characteristics of studied plots.

Country	Plot	Cation status	WRB soil classification	Sand	Clay	Silt	pH	C:N	[Ca]_ex_	[Mg]_ex_	[K]_ex_	[Na]_ex_	[P]_t_
									
									mmol kg^−1^				
Australia	RCR-01	Low	Haplic Cambisol (Dystric, Alumic)	0.61	0.30	0.08	5.0	11.9	8.4	5.1	2.4	0.5	9.4
	DRO-01	High	Haplic Cambisol (Hyperdystric, Alumic, Skeletic)	0.19	0.28	0.54	5.6	16.9	17.9	7.6	0.7	0.6	15.3
Bolivia	LFB-01	Low	Geric Acric Ferrasol (Alumic, Hyperdystric)	0.74	0.20	0.06	4.6	13.0	0.2	0.6	0.7	0.0	3.3
	HCC-22	High	Vetic Nitisol (Hypereutric, Rhodic)	0.66	0.21	0.13	5.8	10.8	36.1	9.3	1.5	0.1	13.3
Ecuador	JAS-02	Low	Haplic Alisol (Hyperdystric, Clayic)	0.42	0.29	0.30	4.8	9.8	13.0	6.2	0.8	0.4	5.4
	BOG-02	High	Haplic Cambisol (Orthoeutric)	0.47	0.30	0.23	4.9	8.0	59.8	16.3	1.4	0.3	13.3
Peru	TAM-07	Low	Haplic Cambisol (Alumic, Hyperdystric, Cromic)	0.47	0.29	0.24	4.2	10.4	0.4	1.0	0.8	0.3	5.8
	CUZ-03	High	Plinthic Cambisol (Orthoeutric)	0.05	0.42	0.52	6.1	7.6	49.5	16.7	2.0	0.2	23.5

*Sand, clay, and silt are expressed in proportions. Soil pH, soil carbon: nitrogen ratio, exchangeable soil cations (viz. calcium [Ca]_ex_, magnesium [Mg]_ex_, potassium [K]_ex_, and sodium [Na]_ex_) and total soil phosphorus [P]_t_ (0 to 0.3 m depth) at the eight study sites.*

### Wood and Leaf Trait Variation Among Contrasting Cation Soil Status

**FIGURE 2:**
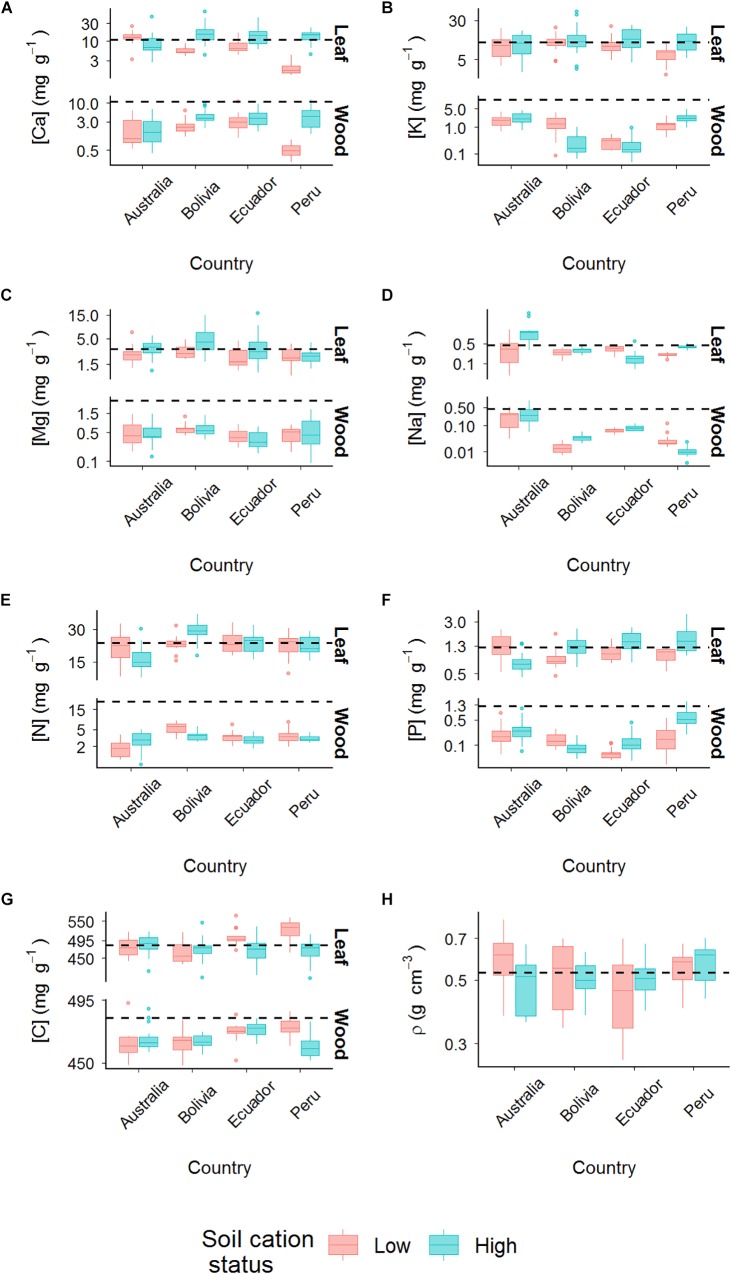
Variation of elements concentration in leaf and wood. **(A)** Calcium; **(B)** potassium; **(C)** magnesium; **(D)** sodium; **(E)** nitrogen; **(F)** phosphorus; **(G)** carbon; **(H)** wood density. Countries are indicated on *x*-axis and plots within each country are labeled as low and high soil cation status (red and blue, respectively). The *y*-axis scales are in log_10_. Dashed lines depict average leaf value of each trait. The average value of leaf trait is also plotted on wood traits plots (dashed lines) to contrast with the wood values. In the wood density graph (ρ), dashed line represents mean value the trait across plots.

Contrasts between the plots in terms of leaf and wood cation, C, N, and P concentrations (hereafter presented as [Element]_L_, in which the concentration of the element is presented in between brackets and subscript L and W designate leaf and wood, respectively) are shown in Figure 2A–G, with Figure 2H further showing wood density variations across plots (actual values and ranges are shown in Supplementary Table S2, and results of tests for significant difference are shown in Supplementary Table S3). Taken together, this shows [Ca]_L_ to vary systematically (though not always significantly) across the Amazon forest plots with, on average, lower concentrations being found in trees from low cation status soils for Bolivia, Ecuador, and Peru. On the other hand, for Australia, [Ca]_L_ were lower for trees growing on the high cation status DRO-01 soil than for the low cation status RCR-01 (Figure 2A). For all four sampling locations, [Ca]_W_ were greater for the high vs. low cation status soils, although again, this difference was not always significant at *p* < 0.05. Within-plot averaged wood calcium concentrations ranged from 17 to 42% of the plot-averaged foliar calcium values.

As for potassium, leaves had much higher [K] than was found in branches. However, as is also clear from Figure 2B, the extent of variation in [K]_L_ between plots was considerably less. On the other hand, wood potassium concentrations varied substantially between locations with the two plots in Ecuador and the low cation status plot in Bolivia having [K]_W_ almost 100 times lower than Australian and Peruvian plots.

In comparison to both potassium and calcium, average [Mg]_W_ were relatively invariant across plots (Figure 2C) and with there being no significant differences for trees growing on soils of low vs. high cation status (*p* = 0.96, Supplementary Table S1). Leaves showed similarly invariant patterns but at concentrations *ca*. five times higher (Figure 2C). In terms of between-plot variation, only the high cation status plot in Bolivia showed significantly higher average [Mg]_L_ values as compared to the low cation status soil (*p* < 0.05; Supplementary Table S1).

Between-plot variations of [Na] in both wood and leaf was considerable, with the Australian plots showing the highest concentrations in both organs. Although this large range of [Na]_W_ variation was mainly driven by the extremely high values in Australian plots, both [Na]_L_ and [Na]_W_ varied substantially across the Amazon Basin sites. For example, the Bolivian high cation status plot had the highest concentrations in wood while the opposite pattern was found in the Peruvian plots. Similarly, whilst [Na]_L_ was highest in high cation status plot in Peru, it was lowest in the Ecuadorian high cation status plot. Both [Na]_L_ and [Na]_W_ differed between Peruvian plots (*p* = 0.03 and *p* < 0.001 for leaves and wood, respectively) with higher foliar concentrations observed for the high cation status CUZ-03 as compared to the lower cation status TAM-07, but with the wood sodium concentrations being higher on the lower cation status TAM-07 soil (Figure 2D).

Nitrogen in leaves varied significantly with soil cation status only for Bolivia (*p* = 0.02, Supplementary Table S1) with the high cation status plot showing higher concentrations (Figure 2E). For woody tissues, nitrogen concentrations showed opposite patterns for Australia vs. Bolivia, with lower [N]_W_ in the low cation status plot in Australia (*p* = 0.01) and higher concentrations in the low cation status plot in Bolivia. Nevertheless, this difference was only marginally significant (*p* = 0.06).

**FIGURE 3:**
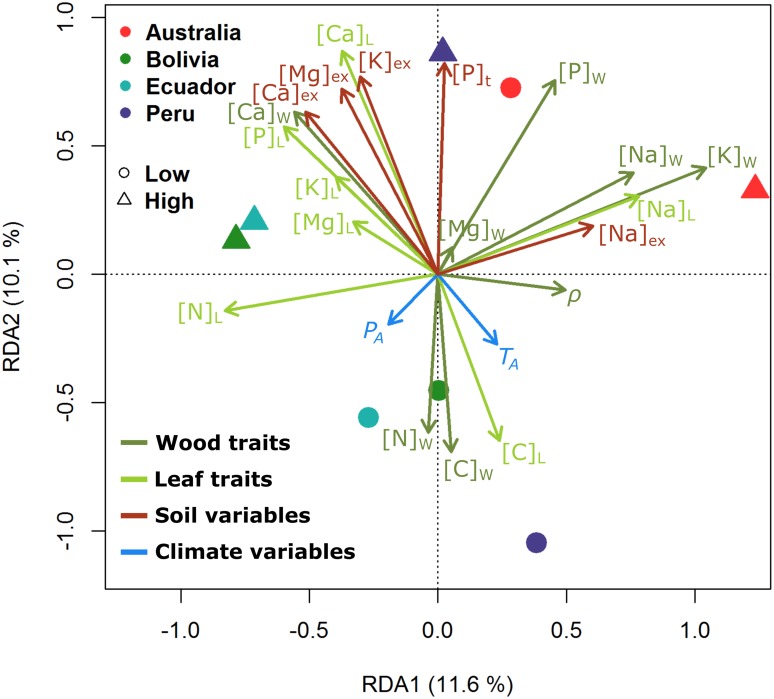
Redundancy analysis triplot (RDA) showing the relationships among sites (circles/triangles), environmental variables (soil properties as red arrows, climate characteristics as blue arrows), and the studied traits (wood as dark green arrows, leaves as bright green arrows). Subscripts W, wood traits; L, leaf traits; ex, soil exchangeable cation, t, total. Different colors for the points depict countries with the contrasting soil cation status plots classified as “Low” and “High” as detailed in Table 1. The percentage of the total variance explained by the first two canonical eigenvalues (RDA1 and RDA2) are indicated in the axes. The length of the arrows is equal to the multiple correlation of that variable with the displayed ordination axes and thus provides an indication of how well the values of the variable are displayed in the biplot of sites and environmental variables. The (cosine of the) angles between the trait and soil/climate variables, and between the trait variables themselves or climate variables themselves reflect their correlations.

The pattern of [P]_W_ variation across plots was broadly similar to that observed for [K]_W_ variation, but across a much lower degree of variability (Figure 2F), and with high cation status plots showing significantly higher [P]_W_ than lower cation status plots only in Peru (*p* < 0.001). Low cation status plots had lower [P]_L_ than their high cation status counterparts in Bolivia, Ecuador and Peru (*p* = 0.01, *p* = 0.03, and *p* < 0.01, respectively), with Australia showing the opposite relationship (*p* < 0.01, Supplementary Table S1).

Carbon varied more amongst foliar tissues than for wood (Figure 2G) with the highest [C]_L_ found in trees from the low cation soil in Peru. For the Ecuador (*p* = 0.02) and Peru plots (*p* < 0.01), [C]_L_ were significantly lower for the high vs. low cation status soils. Carbon in wood varied with soil cation status only for the Peruvian plots (*p* < 0.01), in this case showing a similar pattern to the [C]_L_ (i.e., higher for the lower cation status soil).

Both the highest and lowest wood densities were found in Australia, with mean values varying from 0.49 g cm^−3^ at RCR-01 to 0.61 g cm^−3^ at DRO-01. But at no location did ρ vary with plot cation status at *p* < 0.05 or less (Figure 2H and Supplementary Table S1).

### Contrasting Variations in Wood and Leaf Elemental Concentrations as Related to Soil and Climate

Figure 3 shows the results of the RDA ordination, in which the studied leaf and wood trait characteristics are associated to the soil and climate properties in the plots. For example, although the similar directions of the arrows of [Ca]_L_, [Mg]_L_, and [K]_L_ all indicate reasonably strong associations amongst these three cations as well as with the associated exchangeable soil cation concentration (viz. [Ca]_ex_, [Mg]_ex_, and [K]_ex_, as also evidenced by the similar angles) of the wood cations a similar pattern was only observed for [Ca]_W_. Conversely, within the triplot, [K]_W_ and [K]_ex_ are nearly perpendicular to each other, thus indicating that woody tissue [K] varied across plots more or less independently of any variations in [K]_ex_ or the closely associated [K]_L_. Likewise, the ordination shows that neither [P]_L_ or [P]_W_ show any strong association with total soil phosphorus concentration and with there being little if any relationship between [Mg]_W_ and both [Mg]_L_ and [Mg]_ex_. Figure 3 also shows a markedly different pattern in [Na]_ex_ variability as compared to the other measured exchangeable cations, with [Na]_L_, [Na]_W_, and [K]_W_ all strongly aligning with [Na]_ex_. In addition, Figure 3 indicates that [C]_L_, [C]_W_, and [N_w_] varied across sites in a similar manner to each other and with a negative association with all [Ca]_ex_, [Mg]_ex_, [K]_ex_, and [P]_t_. Wood density did not align closely with any of the studied leaf/wood or soil/climate variables. Neither *P*_A_ nor *T*_A_ was closely associated with the two main axes of trait/environmental variation.

### Wood/Leaf Trait Associations

**FIGURE 4:**
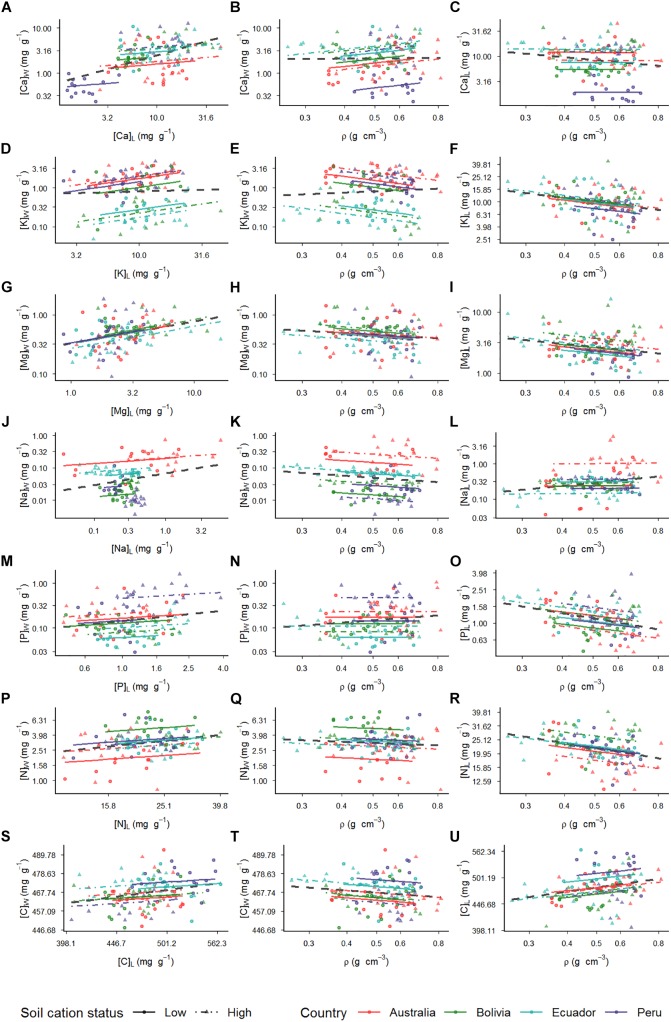
Relationships of wood and leaf traits. Each line of the panel indicates associations with specific cations. First column of the panel depicts plots of cation relations among wood and leaf. Second column depicts plots of cations in wood and wood density relations. Third column has plots of relations between cations in leaves and wood density. Relationships are assessed by using mixed models with plot where tree grows as random effect (Eq. 1). Each location is represented by a different color with plots within location depicting different line types and symbols. The relationship between the traits for each plot is indicated by a different line color and type with the low dashed line depicting the low cation status soils whereas the high cation status soils are represented by the dotted lines. Thicker dashed black line represents ordinary least squares (OLS) model (Eq. 3). Relationships between: **(A)** calcium in wood and leaves; **(B)** calcium in wood and wood density; **(C)** calcium in leaves and wood density; **(D)** potassium in wood and leaves; **(E)** potassium in wood and wood density; **(F)** potassium in leaves and wood density; **(G)** magnesium in wood and leaves; **(H)** magnesium in wood and wood density; **(I)** magnesium in leaves and wood density; **(J)** sodium in wood and leaves; **(K)** sodium in wood and wood density; **(L)** sodium in leaves and wood density; **(M)** phosphorus in wood and leaves; **(N)** phosphorus in wood and wood density; **(O)** phosphorus in leaves and wood density; **(P)** nitrogen in wood and leaves; **(Q)** nitrogen in wood and wood density; **(R)** nitrogen in leaves and wood density; **(S)** carbon in wood and leaves; **(T)** carbon in wood and wood density; **(U)** carbon in leaves and wood density.

For all seven elements studied, Figure 4 shows the relationships between the concentrations in leaf and wood (first column: Panels A, D, G, J, M, P, and S); the relationship between concentrations in wood and the associated wood density (middle column: Panels B, E, H, K, N, Q, and T); and the relationship between foliar concentrations and ρ (third column: Panels C, F, I, L, O, R, and U). Within each panel are shown both the results of the MEM (separate symbols and lines for each plot) and the OLS regression (thicker dashed line): the latter as obtained by simply analyzing all trees together without any consideration of plot location. Comparing the MEM results with the OLS, a significant random intercept was found for all associations with exception of [Mg]_L_ vs. [Mg]_W_ (Supplementary Table S4), but in no case did the MEM analysis also indicate significant random slope effects associated with plot location (exact likelihood ratio test: data not shown).

Nevertheless, many of the MEM within-plot slope estimates differed from those implied by the OLS in terms of statistical significance and, in some cases, in slope as well (Table 3). For example, although the slope and statistical significance of the [Mg]_W_ vs. [Mg]_L_ association as shown in Figure 4G was similar for the MEM (γ_10_ = 0.37 ± 0.10, *p* < 0.001 and OLS (β_1_ = 0.38 ± 0.09, *p* < 0.001), the OLS model suggested leaf-wood nutrient associations not evident the MEM analysis for carbon (Figure 4S), calcium (Figure 4A), sodium (Figure 4J), and phosphorus (Figure 4M), with the OLS β_1_ coefficients in all cases having the steeper slope (Table 3). This contrast was most evident for calcium for which the OLS slope of β_1_ = 0.61 ± 0.09, (*p* < 0.001) was over three times greater than the within-plot slope as inferred from the MEM (γ_10_ = 0.18 ± 0.12, *p* = 0.13). On the other hand, the MEM was able to detect a strong within-plot [K]_W_ vs. [K]_L_ association (γ_10_ = 0.46 ± 0.13, *p* < 0.001) but with this relationship (as shown in Figure 4D) being overlooked the simpler OLS analysis (β_1_ = 0.08 ± 0.21, *p* = 0.69).

In terms of wood nutrient vs. wood density associations significant slopes were found for the [K]_W_ vs. ρ (Figure 4E) when estimated using MEM (γ_10_ = −1.06 ± 0.29, *p* < 0.001) but with the OLS slope estimate of β_0_ = 0.35 ± 0.47 not significantly different from zero (*p* = 0.46). Wood sodium concentrations also declined with wood density (Figure 4K) with the relationship significant at *p* = 0.005 as analyzed by MEM (γ_10_ = −0.65 ± 0.23) but with, despite it being of a nearly identical magnitude, the slope as obtained by the OLS (β_1_ = −0.68 ± 0.48) only being significant at *p* = 0.16.

**Table 3:** Coefficients and intercepts for bivariate relationships of traits using mixed model MEM (Eq. 1) and ordinary least squares OLS (Eq. 3).

	Wood vs. Leaf				Wood vs. Wood density ρ				Leaf vs. Wood density ρ			
	MEM		OLS		MEM		OLS		MEM		OLS	
Element	γ_10_	γ_00_	β_1_	β_0_	γ_10_	γ_00_	β_1_	β_0_	γ_10_	γ_00_	β_1_	β_0_
C	0.03	2.58	**0.07**	2.5	−0.01	2.67	−0.01	2.67	**0.06**	2.7	**0.08**	2.71
Ca	0.18	0.18	**0.61**	−0.2	0.85	0.56	0.04	0.34	−0.02	0.9	−0.56	0.75
K	**0.46**	−0.58	0.08	−0.2	**−1.06**	−0.43	0.35	0.01	**−0.57**	0.83	**−0.63**	0.81
Mg	**0.38**	−0.49	**0.37**	−0.5	−0.41	−0.46	−0.3	−0.43	**−0.52**	0.27	**−0.51**	0.27
N	0.25	0.16	**0.38**	0	−0.2	0.44	−0.16	0.45	**−0.29**	1.25	**−0.36**	1.23
Na	0.16	−1.25	**0.36**	−1.2	**−0.65**	−1.51	−0.68	−1.51	0.08	−0.51	**0.88**	−0.28
P	0.22	−0.85	**0.38**	−0.8	−0.01	−0.84	0.53	−0.67	**−0.47**	−0.09	**−0.65**	−0.14

*In the mixed effect model, sampling plot is used as a random intercept. Bold coefficients depict significant relationships (p < 0.05). γ_i_ = slope as presented in model of Eq. 1; γ_00_ = intercept as presented in model of Eq. 1; β_1_ = slope as presented in models of Eq. 3; β_0_ = intercept as presented in model of Eq. 3.*

For the leaf element vs. ρ associations, all were negative for both the MEM and OLS analyses except for the ρ vs. [C]_L_ association (Figure 4U), for which the MEM slope estimate (γ_10_ = 0.06 ± 0.03, *p* = 0.024) was virtually identical to that from the OLS (β_1_ = −0.08 ± 0.03, *p* = 0.005). As detailed in Table 3 strong similarities between the MEM and OLS estimates were also observed for the associations of ρ with [K]_L_ (Figure 4F), [Mg]_L_ (Figure 4I), [P]_L_ (Figure 4O), and [N]_L_ (Figure 4P). By contrast for the association between [Na]_L_ and ρ (Figure 4L), the MEM suggested no relationship between the two traits (γ_10_ = 0.08 ± 0.26, *p* = 0.75) but with a 10-fold higher highly significant OLS estimate obtained from the same data set (β_1_ = 0.87 ± 0.33, *p* = 0.008). There was no strong association between ρ and [Ca]_L_ inferred by either of the models (*p* = 0.09 and *p* = 0.92 for the MEM and OLS, respectively).

## Discussion

### Inter-Organ Nutrient Associations

The differing patterns of variations in leaf and wood nutrients in relation to soils and climate as indicated by Figure 3 suggests that in many cases climate and/or soils may be influencing the concentrations of different elements in different ways. This has obvious implications for the construction and interpretation of leaf vs. wood scaling relationships. For example, with both [Ca]_W_ and [Ca]_L_ varying with soil exchangeable [Ca] in a broadly similar way (Figure 3) then it is not surprising that when data are simply pooled across plots and analyzed by OLS analysis that a very strong association between [Ca]_L_ and [Ca]_W_ emerges (Table 3, Supplementary Table S5, and Figure 4A). But, as demonstrated by our MEM analysis, this strong OLS correlation also occurs despite there actually being no (within-tree) association between [Ca]_W_ and [Ca]_L_ within individual plots (Table 3). This difference demonstrates the potential confusions with conclusions inferred apparently at the individual tree or species level actually being based on relationships that exist almost entirely only at the plot level, a case of the so-called “ecological fallacy” (Robinson, 1950; Snijders and Bosker, 2012). This also seems to be the case for wood and leaf for [Ca], [N], and [P], with no within-plot associations detected through the MEM despite wood and leaf concentrations varying with soil properties in broadly similar manners.

Interestingly, the opposite pattern was observed in our case for potassium with the positive [K]_W_ vs. [K]_L_ observed for trees within individual plots as detected by the MEM analysis not detected by the OLS model. We refer to this phenomenon as “environmental obfuscation” and in this case the contrast arises because, although there was not that much variation in the range of [K]_L_ observed across the different plots, the average [K]_W_ varied substantially with plot location. This was also evidenced by the more than 10-fold variation in random intercept values, but with the slope of relationships between [K]_W_ vs. [K]_L_ within each plot being statistically similar (Figure 4D).

### Wood Density-Branch Nutrient Associations

Functioning as storage cells for nutrients, carbohydrates and water, parenchyma are generally considered the most metabolically active cells in wood (Morris et al., 2018). Thus, with some studies having associated a lower ρ with higher parenchyma content in wood (Borchert and Pockman, 2005; Zheng and Martínez-Cabrera, 2013) a negative relationship between metabolic and/or osmotically important nutrients and ρ might reasonably be anticipated. Nevertheless, of the seven elements tested, a negative relationship with ρ was only found for [K]_W_ and [Na]_W,_ and again with “environmental obfuscation” occurring as this relationship was only found when using the MEM model.

For potassium, this negative relationship within individual plots might be due to its important role in wood formation (Fromm, 2010) with lower [K] in woody tissues also having a negative effect on poplar vessel size and wood production (Langer et al., 2002). With lower ρ also reflecting increased tree stem diameter growth (Poorter et al., 2008) and vessel size (Poorter et al., 2010; Fortunel et al., 2013) a mechanistic link between ρ and K, therefore, seems reasonable. Indeed, working across the Amazon Basin, Quesada et al. (2012) reported lower mean ρ on sites with higher [K]_ex_. Moreover, given that K and Na have similar physicochemical properties (Benito et al., 2014) it seems likely that Na can serve as an osmoticum in plants and therefore substitute for potassium at low K availability (Kronzucker and Britto, 2011), or even in addition to K (Battie-Laclau et al., 2014). The role of these monovalent cations in alleviating the impacts of catastrophic cavitation on xylem conduits by chemically interacting with the pectic polysaccharides of the bordered pit membranes has already been noted. Considering the lower hydraulic safety that many low-ρ species show (Preston et al., 2006; Pratt et al., 2007), the higher concentration of these cations in woody tissues of species with a lower ρ could indicate an evolutionary strategy of plants to cope with their anatomical constraints that enhance embolism susceptibility (e.g., presenting larger vessels) by increasing water conductivity within their xylem. Axial parenchyma cells are responsible for loading the xylem vessels with these cations (de Boer and Wegner, 1997) and higher axial parenchyma fraction in the woody tissues are associated with a lower ρ (Zheng and Martínez-Cabrera, 2013).

### Wood Density-Leaf Nutrient Associations

Using the same plant material as was used here Patiño et al. (2012) have already reported and discussed negative relationships of ρ with all of [N]_L_, [P]_L_, and [K]_L_ as so with these not being considered in any detail here; especially as there was little difference between the MEM and OLS analyses (Table 3). It may, however, be worth noting that the Patiño et al. (2012) study looked at leaf mass per unit area (*M*_a_) as well thus finding a positive relationship with ρ where genotype was the primary source of variation. This has also been found by other workers working at single sites (Bucci et al., 2004; Ishida et al., 2008; Meinzer et al., 2008) and has been taken to suggest that – at least within the same site – trees with a higher ρ produce higher *M*_a_ leaves which can then be sustained through more severe water deficits due to their higher resistance to cavitation. Conversely, although wood densities hardly varying with precipitation regime across the Amazon Basin (Patiño et al., 2012), both Fyllas et al. (2009) and Patiño et al. (2012) reported a significant sampling location dependent component of *M*_a_ which, unlike ρ shows a clear positive association with *P*_A_. This would then seem to be another case where the driving forces underlying the nature of bivariate correlations within a plot are fundamentally different to those operating when trees in different plots are compared.

## Conclusion

There are systematic variations in tropical tree wood nutrient concentrations which do not necessarily vary with sampling location in the same way as is observed for leaf nutrient concentrations. This means that the relative concentrations of the same elements in these two different organs vary from site to site. Even with such differences (as evidenced through there being a significant random intercept variation when analyzed in a MEM framework), when expressed on a log-log basis, the slope of many bivariate relationships is surprisingly consistent across plots. This is despite different intercepts and means that the nature of the observed within-plot covariations can be fundamentally different to the underlying differences between plots. Thus, conventional OLS analyses which pool data across plots are likely to give rise to erroneous conclusions as to the nature and ecological significance of any associations inferred.

In this study the MEM vs. OLS difference was especially pronounced for wood potassium concentrations, for which there were strong associations with both leaf potassium concentrations and wood density within, but not across, plots. This “environmental obfuscation” was attributable to wood [K] showing a much larger variation across different plots than observed for either foliar [K] or wood density. The systematic cross-organ association for [K] as well as the association of this element with wood density reinforces the relevance of this nutrient in regulating tree physiology, which has been vastly overlooked in ecological studies (Lloyd et al., 2015; Sardans and Peñuelas, 2015; Schrodt et al., 2015).

## Data Availability

The datasets generated for this study are available on request to the corresponding authors.

## Author Contributions

DL-M and JL designed the study, provided leaf and wood data, and wrote the manuscript. DL-M, EH-W, and SS developed the laboratorial protocols and analyzed wood samples. DL-M and FI conducted the field work in Australia. CQ provided the South American soil data. DL-M carried out all the statistical analyses with substantial inputs from JL. All authors edited the manuscript and approved its current version.

## Conflict of Interest Statement

The authors declare that the research was conducted in the absence of any commercial or financial relationships that could be construed as a potential conflict of interest.
