# Supplementary material for: Tropical Tree Branch-Leaf Nutrient Scaling Relationships Vary With Sampling Location
Source: Front Plant Sci. 2019 Jul 5;10:877. doi: 10.3389/fpls.2019.00877 (PMC6625373; doi:10.3389/fpls.2019.00877)
Supplement: Supplementary file 1 [file Data_Sheet_1.docx]

Table S1. Number of sampled species in each studied site

|  | Ecuador | | Peru | | Bolivia | | Australia | |
| --- | --- | --- | --- | --- | --- | --- | --- | --- |
| Species | BOG-02 | JAS-02 | CUZ-03 | TAM-07 | HCC-22 | LFB-01 | DRO-01 | RCR-01 |
| *Acacia celsa* | - | - | - | - | - | - | - | 1 |
| *Agathis microstachya* | - | - | - | - | - | - | - | 1 |
| *Alchornea glandulosa* | - | - | - | - | 1 | - | - | - |
| *Aleurites rockinghamensis* | - | - | - | - | - | - | - | 4 |
| *Alphitonia petriei* | - | - | - | - | - | - | - | 2 |
| *Aniba guianensis* | - | - | 1 | - | - | - | - | - |
| *Aniba taubertiana* | - | - | 1 | - | - | - | - | - |
| *Aniba terminalis* | - | - | 1 | - | - | - | - | - |
| *Apeiba aspera* | - | 1 | - | - | - | - | - | - |
| *Argyrodendron peralatum* | - | - | - | - | - | - | 1 | - |
| *Bauhinia brachycalyx* | 1 | - | - | - | - | - | - | - |
| *Bertholettia excelsa* | - | - | - | 1 | - | - | - | - |
| *Brosimum acutifolium* | - | - | - | - | - | 1 | - | - |
| *Byrsonima crispa* | - | - | - | - | 1 | - | - | - |
| *Calophyllum brasiliense* | - | - | - | 1 | - | - | - | - |
| *Cardwellia sublimis* | - | - | - | - | - | - | 2 | 1 |
| *Castanospermum australe* | - | - | - | - | - | - | - | 1 |
| *Celtis paniculata* | - | - | - | - | - | - | 1 | - |
| *Chionanthus implicatus* | - | 1 | - | - | - | - | - | - |
| *Clarisia biflora* | - | - | 1 | - | - | - | - | - |
| *Cleistanthus myrianthus* | - | - | - | - | - | - | 1 | - |
| *Coccoloba densifrons* | 1 | - | - | - | - | - | - | - |
| *Croton tessmannii* | 1 | - | - | - | - | - | - | - |
| *Cryptocarya mackinnoniana* | - | - | - | - | - | - | 1 | - |
| *Dendropanax caucanus* | - | 1 | - | - | - | - | - | - |
| *Drypetes amazonica* | - | - | 2 | - | - | - | - | - |
| *Dysoxylum papuanum* | - | - | - | - | - | - | 1 | - |
| *Dysoxylum pettigrewianum* | - | - | - | - | - | - | 1 | - |
| *Elaeocarpus angustifolius* | - | - | - | - | - | - | - | 1 |
| *Endiandra microneura* | - | - | - | - | - | - | 4 | - |
| *Erisma uncinatum* | - | - | - | - | 1 | 1 | - | - |
| *Ficus maxima* | - | - | - | - | 2 | - | - | - |
| *Flindersia brayleyana* | - | - | - | - | - | - | - | 1 |
| *Ganophyllum falcatum* | - | - | - | - | - | - | 1 | - |
| *Geissois biagiana* | - | - | - | - | - | - | - | 1 |
| *Grias neuberthii* | 1 | - | - | - | - | - | - | - |
| *Guarea grandifolia* | 1 | - | - | - | - | - | - | - |
| *Hasseltia floribunda* | - | 1 | - | - | - | - | - | - |
| *Helicostylis tomentosa* | - | - | - | 1 | - | 1 | - | - |
| *Hyeronima oblonga* | - | - | - | - | - | 3 | - | - |
| *Hymenaea parvifolia* | - | - | - | 1 | - | - | - | - |
| *Ilex inundata* | - | 1 | - | - | - | - | - | - |
| *Inga bourgonii* | - | - | 1 | - | - | - | - | - |
| *Inga coriacea* | - | 1 | - | - | - | - | - | - |
| *Inga fagifolia* | - | 1 | - | - | - | - | - | - |
| *Inga laurina* | - | - | 1 | - | - | - | - | - |
| *Inga marginata* | - | - | - | - | 1 | - | - | - |
| *Iryanthera juruensis* | 1 | - | - | - | - | - | - | - |
| *Iryanthera laevis* | - | - | - | 1 | - | - | - | - |
| *Jacaranda copaia* | - | - | - | 1 | - | - | - | - |
| *Laetia procera* | - | 1 | - | - | - | - | - | - |
| *Lunania parviflora* | 1 | - | - | - | - | - | - | - |
| *Melicope elleryana* | - | - | - | - | - | - | - | 3 |
| *Metrodorea flavida* | - | - | - | - | 4 | - | - | - |
| *Miconia dolichorrhyncha* | - | - | - | 1 | - | - | - | - |
| *Miconia multiflora* | - | - | - | - | - | 1 | - | - |
| *Minquartia guianensis* | - | - | 1 | - | - | - | - | - |
| *Musgravea heterophylla* | - | - | - | - | - | - | 2 | - |
| *Myristica globosa* | - | - | - | - | - | - | 1 | - |
| *Myroxylon balsamum* | - | - | 1 | - | - | - | - | - |
| *Nectandra crassiloba* | - | 1 | - | - | - | - | - | - |
| *Osteophloeum platyspermum* | - | 1 | - | - | - | - | - | - |
| *Pachira aquatica* | 1 | - | - | - | - | - | - | - |
| *Pourouma guianensis* | - | - | - | - | 3 | 1 | - | - |
| *Pourouma minor* | - | - | - | 4 | - | - | - | - |
| *Pourouma petiolulata* | 1 | - | - | - | - | - | - | - |
| *Pourouma tomentosa* | - | 1 | - | - | - | - | - | - |
| *Pouteria ephedrantha* | - | - | 1 | - | - | - | - | - |
| *Pouteria glomerata* | - | - | - | - | 1 | - | - | - |
| *Pouteria torta* | - | - | 1 | - | - | - | - | - |
| *Protium fimbriatum* | - | 1 | - | - | - | - | - | - |
| *Pseudolmedia laevis* | 2 | - | 2 | - | - | 3 | - | - |
| *Pseudolmedia macrophylla* | - | - | - | - | - | 2 | - | - |
| *Quararibea bicolor* | - | - | 1 | - | - | - | - | - |
| *Quararibea cordata* | 1 | - | - | - | - | - | - | - |
| *Quararibea obliquifolia* | 1 | - | - | - | - | - | - | - |
| *Quararibea wittii* | - | - | 2 | - | - | - | - | - |
| *Rinorea apiculata* | 1 | - | - | - | - | - | - | - |
| *Sclerolobium bracteosum* | - | - | - | 1 | - | - | - | - |
| *Sloanea eichleri* | - | - | - | - | - | 1 | - | - |
| *Sterculia colombiana* | - | 1 | - | - | - | - | - | - |
| *Syzygium gustavioides* | - | - | - | - | - | - | 1 | - |
| *Syzygium kuranda* | - | - | - | - | - | - | 1 | - |
| *Syzygium sayeri* | - | - | - | - | - | - | 1 | - |
| *Tachigali polyphylla* | - | - | - | 1 | - | - | - | - |
| *Tetragastris altissima* | - | - | - | 1 | - | - | - | - |
| *Trichilia pleeana* | 1 | - | - | - | - | - | - | - |
| *Turpinia occidentalis* | - | - | 1 | - | 1 | - | - | - |
| *Unonopsis floribunda* | 1 | - | - | - | - | - | - | - |
| *Virola sebifera* | - | - | - | 1 | - | - | - | - |

|  |  | **C (mg g^-1^)** | | **Ca (mg g^-1^)** | | **K (mg g^-1^)** | | **Mg (mg g^-1^)** | |
| --- | --- | --- | --- | --- | --- | --- | --- | --- | --- |
| Country | Soil cation status | Leaf | Wood | Leaf | Wood | Leaf | Wood | Leaf | Wood |
| Australia | Low | 478.3^a^  (442.7-519.4) | 462.8^a^  (448.7-492.9) | 13.2^a^  (3.2-24.5) | 2.2^a^  (0.6-6.3) | 10.1^a^  (3.9-20.7) | 1.8^a^  (0.7-3.8) | 2.6^a^  (1.2-6.6) | 0.5^a^  (0.2-1.4) |
|  | High | 482.5^a^  (419.23-518.5) | 466.5^a^  (457.4-488.5) | 10.5^a^  (2.7-44.2) | 2.2^a^  (0.4-6.7) | 10.9^a^  (2.8-19.0) | 2.3^a^  (0.8-4.2) | 3.3^a^  (1.1-5.7) | 0.6^a^  (0.1-1.4) |
| Bolivia | Low | 463.9^a^  (435.1-516.62) | 464.3^a^  (448.0-480.2) | 5.5^a^  (4.0-8.1) | 2.3^a^  (1.2-6.2) | 11.3^a^  (4.6-21.4) | 1.5^a^  (0.1-3.6) | 2.7^a^  (1.9-4.9) | 0.6^a^  (0.4-0.9) |
|  | High | 472.2^a^  (404.9-544.4) | 464.8^a^  (455.5-471.6) | 15.6^b^  (4.1-36.8) | 4.3^b^  (2.0-9.3) | 12.8^a^  (3.3-44.2) | 0.3^b^  (0.1-1.0) | 4.8^b^  (1.7-12.8) | 0.7^a^  (0.3-1.4) |
| Ecuador | Low | 504.0^a^  (468.9-566.5) | 471.6^a^  (451.6-484.5) | 8.3^a^  (4.3-16.5) | 3.5^a^  (1.1-10.9) | 10.3^a^  (4.9-23.0) | 0.3^a^  (0.1-0.5) | 2.1^a^  (1.1-4.5) | 0.4^a^  (0.2-0.8) |
|  | High | 468.1^b^  (410.3-533.2) | 473.1^a^  (463.0-481.6) | 15.9^b^  (5.9-43.6) | 4.3^a^  (1.6-9.2) | 14.6^a^  (4.3-24.5) | 0.3^a^  (0.1-0.9) | 4.1^a^  (1.0-16.4) | 0.4^a^  (0.2-0.7) |
| Peru | Low | 521.8^a^  (462.9-559.7) | 475.5^a^  (461.7-486.6) | 1.9^a^  (1.2-4.1) | 0.5^a^  (0.2-1.0) | 6.5^a^  (2.5-10.4) | 1.3^a^  (0.4-2.7) | 2.2^a^  (0.9-3.9) | 0.5^a^  (0.2-0.8) |
|  | High | 469.9^b^  (403.0-511.4) | 460.62^b^  (451.5-479.3) | 14.0^b^  (4.3-22.6) | 4.8^b^  (1.4-12.4) | 12.0^b^  (5.4-22.2) | 2.3^a^  (1.0-4.7) | 2.34^a^  (1.21-4.1) | 0.6^a^  (0.1-1.8) |

Table S2. Arithmetic average values and range between brackets of each assessed trait per plot. Significant differences of mean values between plots within each country (*p* < 0.05) are indicated by different superscript letters a and b and were obtained with the Tukey’s ‘Honest Significant Difference’ method. Plots with non-significant differences show same superscript letter.

**Table S2. Continued**

|  |  | **N (mg g^-1^)** | | **Na (mg g^-1^)** | | **P (mg g^-1^)** | | ***ρ* (g cm^-3^)** |
| --- | --- | --- | --- | --- | --- | --- | --- | --- |
| Country | Soil cation status | Leaf | Wood | Leaf | Wood | Leaf | Wood | Wood |
| Australia | Low | 21.9^a^  (11-3.9) | 1.9^a^  (1.0-3.8) | 0.39^a^  (0.04-1.51) | 0.21^a^  (0.03-0.43) | 1.4^b^  (0.5-2.3) | 0.23^a^  (0.06-0.78) | 0.49^a^  (0.36-0.67) |
|  | High | 16.8^a^  (10.9-30.3) | 3.0^b^  (0.7-4.9) | 1.54^b^  (0.3-5.85) | 0.32^a^  (0.06-0.91) | 0.8^a^  (0.4-1.4) | 0.30^a^  (0.07-1.04) | 0.61^b^  (0.38-0.81) |
| Bolivia | Low | 22.3^a^  (15.4-32.3) | 5.4^a^  (3.0-8.1) | 0.25^a^  (0.12-0.36) | 0.02^a^  (0.01-0.03) | 0.9^a^  (0.5-2.0) | 0.13^a^  (0.07-0.22) | 0.5^a^  (0.38-0.62) |
|  | High | 28.4^b^  (17.1-39.9) | 3.5^a^  (2.6-5.9) | 0.3^a^  (0.19-0.41) | 0.03^b^  (0.02-0.06) | 1.3^b^  (0.6-2.2) | 0.09^a^  (0.04-0.18) | 0.53^a^  (0.34-0.7) |
| Ecuador | Low | 23.0^a^  (17.0-34.4) | 3.5^a^  (2.0-6.5) | 0.32^a^  (0.16-0.42) | 0.07^a^  (0.04-0.08) | 1.1^a^  (0.7-1.6) | 0.06^a^  (0.04-0.11) | 0.52^a^  (0.39-0.67) |
|  | High | 23.5^a^  (17.7-33.0) | 2.9^a^  (1.8-4.4) | 0.16^b^  (0.06-0.6) | 0.08^a^  (0.06-0.12) | 1.6^b^  (0.7-2.5) | 0.13^a^  (0.04-0.35) | 0.46^a^  (0.26-0.7) |
| Peru | Low | 21.8^a^  (11.8-30.6) | 3.7^a^  (2.0-7.4) | 0.2^a^  (0.14-0.25) | 0.03^a^  (0.01-0.12) | 1.0^a^  (0.5-1.2) | 0.19^a^  (0.03-0.57) | 0.58^a^  (0.43-0.7) |
|  | High | 21.5^a^  (15.4-28.7) | 3.1^a^  (2.4-4.3) | 0.39^b^  (0.27-0.51) | 0.01^b^  (0-0.02) | 1.7^b^  (0.9-3.8) | 0.62^b^  (0.2-1.58) | 0.56^a^  (0.4-0.67) |

**Table S3.** Comparison of element concentrations in wood and leaf in different soil cation status. Country was used as a co-variable in ANOVA. *p* values indicate significance (*p* < 0.05) of overall arithmetic mean differences of each trait

| **Element** | **Wood** | **Leaf** |
| --- | --- | --- |
|  | *P* value | *P* value |
| C | 0.113 | **<0.001** |
| Ca | **<0.001** | **<0.001** |
| K | 0.209 | **0.006** |
| Mg | 0.957 | **<0.001** |
| N | 0.336 | 0.958 |
| Na | 0.153 | **<0.001** |
| P | **<0.001** | **0.004** |
| WD | 0.425 | - |

**Table S4.** *P* values based on 10000 simulated values for the restricted likelihood ratio test obtained by using the exactLRT function from package RLRsim (Scheipl et al., 2008).

| Element | Wood *vs* Leaf | Wood *vs ρ* | Leaf *vs* *ρ* |
| --- | --- | --- | --- |
| C | <0.001 | <0.001 | <0.001 |
| Ca | <0.001 | <0.001 | <0.001 |
| K | <0.001 | <0.001 | <0.001 |
| Mg | 0.071 | 0.034 | <0.001 |
| N | <0.001 | <0.001 | 0.012 |
| Na | <0.001 | <0.001 | <0.001 |
| P | <0.001 | <0.001 | <0.001 |

**Table S5**. Coefficients and intercepts of trait associations using plot as random effect which include plot average trait value (i.e. between plot coefficient, Eqn 2) are presented. Bold coefficients depict significant relationships (*p* < 0.05).

| Mixed model results with between group regression coefficient | | | | | | | | | |
| --- | --- | --- | --- | --- | --- | --- | --- | --- | --- |
|  | Wood x Leaf | | | Wood x *ρ* | | | Leaf x *ρ* | | |
| Elements | $\gamma_{10}$ | $\gamma_{00}$ | $\gamma_{01}$ | $\gamma_{10}$ | $\gamma_{00}$ | $\gamma_{01}$ | $\gamma_{10}$ | $\gamma_{00}$ | $\gamma_{01}$ |
| C | 0.02 | 2.17 | 0.16 | -0.01 | 2.67 | 0.16 | **0.06** | 2.73 | 0.12 |
| Ca | 0.08 | -0.47 | **0.77** | **0.80** | -0.58 | -4.10 | 0.00 | 0.03 | -3.15 |
| K | **0.47** | 1.71 | -2.23 | **-1.09** | 1.49 | 6.98 | **-0.52** | 0.66 | -0.66 |
| Mg | **0.40** | -0.40 | -0.20 | **-0.48** | -0.21 | 0.96 | **-0.54** | 0.35 | 0.29 |
| N | 0.24 | -0.20 | 0.28 | -0.20 | 0.59 | 0.56 | **1.10** | -0.27 | -0.56 |
| Na | 0.14 | -0.94 | 0.67 | **-0.65** | -1.44 | 0.28 | 0.03 | 0.81 | **4.81** |
| P | 0.21 | -0.88 | 0.47 | -0.04 | 0.00 | 3.06 | **-0.44** | -0.39 | -1.11 |
